# Vaccination modality and injection site influence the immune response and protection against sea lice *Lepeophtheirus salmonis* infestation in Atlantic salmon (*Salmo salar*)

**DOI:** 10.1016/j.bbrep.2026.102573

**Published:** 2026-04-08

**Authors:** Elisabeth Gislefoss, Amr Gamil, Øystein Evensen

**Affiliations:** Norwegian University of Life Sciences, Faculty of Veterinary Medicine, PO Box 5003, Ås, N-1432, Norway

## Abstract

Sea lice (*Lepeophtheirus salmonis*) infestations represent a challenge to Atlantic salmon (*Salmo salar* L.) aquaculture. We evaluated recombinant peroxinectin-like proteins (LsPxtl-1 and LsPxtl-2) and whole sea lice (WSL) crude proteins as vaccine antigens, administered intraperitoneally (i.p.) or via a combination of i.p./subcutaneous fin-base injection (IP–F). Fish were immunized, boosted, smoltified, and challenged with copepodites, and systemic (plasma) and mucosal (mucus) antibody responses were assessed alongside lice burden. LsPxtl-1 (p < 0.05) reduced lice counts compared to PBS control but not against the empty plasmid PBS group. Elevated systemic IgM/IgT and IgT responses were observed, but levels did not correlate with lice reduction. Mucus antibody responses were generally weak, but suggest IgT may contribute to local protection, particularly in the IP-F group. Results demonstrate that antigen source and vaccine modality influence protection, and protective immunity likely involves additional mechanisms beyond measurable antibodies. Further optimization of antigens, formulations, and delivery strategies is required to achieve reliable vaccine-based control of salmon lice in aquaculture.

## Highlights

• Recombinant LsPxtl-1 and intraperitoneal + fin-base delivery reduced lice counts.
• Systemic antibodies rose after vaccination but did not track lice burden.
• Mucosal antibodies were weak overall, higher with fin-base delivery at low lice.
• Antigen choice and injection site shaped immune responses and protection.
• Results indicate non-antibody mechanisms and a need for optimization.

## 1 Introduction

Lice infestations in salmon farms have a substantial economic impact on the Norwegian aquaculture industry, and the associated economic losses, mainly for treatment, were estimated at around 10.8-19 billion NOK in 2021 [1]. Various treatment methods, including chemical, mechanical, thermal, freshwater, biological, and functional feed treatments, are currently employed to control lice infestations on salmon farms. Chemical treatment was the primary method until 2015; however, the rapid development of resistance to available products has rendered it ineffective. As a result, mechanical, thermal, and freshwater treatment methods have become more widespread. These methods require more fish handling, which can cause stress, increase the risk of wounds, and lead to suboptimal water quality. At the same time, the methods are less effective, especially on the attached lice [[2], [3], [4], [5], [6], [7], [8]]. Currently, no effective treatment methods provide adequate protection from lice infestations. Therefore, alternative methods, such as vaccination, are strongly needed.

Earlier studies have shown that Atlantic salmon develop antibodies against the salmon louse after a natural infestation. However, the antibody levels are low and do not provide adequate protection against reinfection [9]. Nevertheless, this observation indicates the possibility of protection if the “correct” type of immune response is developed. Understanding the host's underlying immune responses after immunization and/or infestation with salmon lice is crucial for identifying the optimal method that provides effective protection.

In Atlantic salmon, three immunoglobulin isotypes, IgM, IgD, and IgT, have been detected [10]. IgM is the dominant systemic antibody and is also detected in the mucus [11]. The function of IgD remains unclear, although it has been suggested to play a role in inflammatory responses [12,13]. IgT, first described as IgZ, was discovered in 2005 [14,15] and has since been shown to have a functional importance in the mucosa of teleost fish [16]. Under non-reduced conditions, IgT has a molecular weight of approximately 180 kDa, whereas the heavy and light chains have been detected at 75 kDa and 25 kDa, respectively [16,17]. IgT responses have been documented in several mucosal tissues, including the gut, gills, skin, and olfactory organs. Dong et al. [18] documented a strong IgT response in the nasopharynx-associated lymphoid tissue in rainbow trout following infestation with *Flavobacterium columnare* [18], and elevated IgT levels were detected in gut mucus during infection with the protozoan parasite *Ceratomyxa shasta* [16].

Further evidence of IgT's role in mucosal parasite immunity was presented in IgT-depletion experiments, where rainbow trout lacking IgT showed increased susceptibility to the parasitic ciliate *Ichthyophthirius multifiliis* (Ich), dysbiosis, tissue damage, and impaired IgM responses. [17]. IgT-mediated responses have also been described in gills and skin mucosa following Ich infestation [11,19]. However, the involvement of IgT in the response against the ectoparasitic salmon louse is unknown, and its role in protecting salmon lice after vaccination and infestation remains to be understood. Vaccination modalities may influence the immune response, and external and internal factors, such as antigen concentration, temperature, and stress tolerance, may also affect vaccine efficacy [20].

Vaccination by injection is the most common method used in Atlantic salmon, giving the best control over individual antigen doses. Injection is most commonly administered intraperitoneally as a protein with an adjuvant formulation or intramuscularly as a DNA vaccine. The fish should weigh at least 15 g; however, the method causes stress and increases handling costs. Nevertheless, this vaccination route is usually more efficient and can provide long-term protection [21]. However, several other administration methods are also used. Oral vaccination is attractive because it is less stressful for fish, can be easily administered, and is suitable for both small and large fish. However, it often lacks efficacy and provides short-term protection, making it difficult to ensure equal uptake [22]. Immersion vaccines can also be administered to very small, yet immunocompetent, fish; however, this method can cause greater stress. Antigens are taken up through the skin, gills, and gut, leading to immune responses.

Alternative delivery methods, such as subcutaneous injection at the base of the dorsal fin, are not well studied. However, studies in teleosts have shown that fin tissue can mount a local immune response following intra-fin administration, including the recruitment of granulocytes, increased phagocytic activity, and a detectable respiratory burst after zymosan injection [23]. A similar study testing Ich antigens via intra-fin administration in ginbuna crucian carp showed increased local responses of CD8^+^ and CD4^+^ T cells [24]. Fins are covered by mucosal epidermis like the skin, and therefore part of the skin-associated lymphoid tissue (SALT), which contains immune-competent cells capable of responding to antigenic stimulation [25]. In Atlantic salmon, studies have shown that salmon lice preferentially attach to dorsal surfaces, including the fin region [26]. Together, these findings support the dorsal fin base as a relevant delivery site for a salmon lice vaccine.

Several recent vaccine studies against salmon lice infestation have assessed different antigen types, including polypeptides, recombinant chimeric antigens, and a recombinant gut protein. A large-scale sea-cage trial with a polypeptide-based vaccine showed only moderate reductions in lice numbers [27]. In contrast, a recombinant gut protein vaccine (P33) reduced lice burden in a laboratory challenge and elicited antigen-specific IgM [28]. Moreover, a promising P0-based chimeric vaccine induced strong early protection under controlled laboratory conditions, although the effect declined over time [29]. To date, no commercially effective salmon lice vaccines exist, underlining the need to better understand the immune response mechanisms influenced by vaccine modalities and delivery methods. In our previous study [30], we identified and characterized two peroxinectin-like proteins (LsPxtl-1 and LsPxtl-2), which are heme peroxidases associated with immune-related functions, making these proteins relevant target antigens for a salmon lice vaccine. Both antigens were previously tested as DNA plasmid vaccines delivered intramuscularly, and the LsPxtl-1-based vaccine was shown to provide some protection.

In general, few studies have attempted to address the generation of the different types of antibody responses seen in naïve fish after infestations and their roles in protection against various pathogens. Although knowledge of the importance of antibody responses against salmon louse infestations is growing, the available data remain scarce, and many hypotheses and questions remain unanswered. Generating knowledge about responses induced by different vaccine modalities and delivery methods will aid in understanding the optimal responses required for protection against lice infestation and, subsequently, in developing efficacious vaccines. In this study, the recombinant antigens (LsPxtl-1 and LsPxtl-2) were administered intraperitoneally (ip) only, while the whole sea lice (WSL) antigens were delivered by either ip alone or by a combination of ip and subcutaneous at the base of the dorsal fin (ip + fin) route. We were interested in assessing how antigen source and vaccination modalities influence the magnitude and type of local antibody response and the level of protection against salmon lice infestation.

## 2 Materials and methods

### 2.1 Salmon lice (*L. salmonis*) and Atlantic salmon (*Salmo salar*)

The lice used in the experiments (LsGulen) were reared on Atlantic salmon, as described by Hamre et al. [31] at the Lice Lab, Sea Lice Research Centre, University of Bergen. Salmon (purchased from the Industry Laboratory, Bergen) were fed a commercial diet and maintained in seawater with a salinity of 34.5 ppt and a temperature of 9 ± .5 °C. Eggs, nauplii, and copepodids were kept in incubators containing seawater with a continuous flow-through system, separate from the host [31]. The experiments performed on live animals were approved by the Norwegian Food Safety Authority and conducted in accordance with Norwegian animal welfare legislation (FOTS ID 8589).

### 2.2 RNA extraction (isolation) and cDNA synthesis

RNA extraction was performed using formalin-fixed adult lice, comprising one female and two males. The lice were added to Eppendorf tubes containing 1 mL ISol-RNA Lysis reagent (5Prime). The parasites were homogenized using the MP FastPrep24 with stainless steel beads. Chloroform (.2 mL) was added to facilitate phase separation of the homogenates, following centrifugation at 12000 rpm for 10 min at 4 °C. The aqueous phase was collected and applied to a gDNA eliminator column using the RNeasy Plus Mini Kit (Qiagen, Hilden, Germany) to remove genomic DNA. The extraction procedure for the RNeasy Plus Mini Kit, as provided by the manufacturer, was then followed. RNA concentrations were measured using a Nanodrop ND-1000 spectrophotometer (Thermo Fisher Scientific, Massachusetts, USA). The cDNA was synthesized from RNA using the Transcription First Strand cDNA Synthesis Kit (Roche, Basel, Switzerland) following the manufacturer's protocol.

### 2.3 Preparation of the antigens LsPxtl1 and LsPxtl2

The preparation of the peroxinectin-like proteins LsPxtl-1 and LsPxtl-2 has been described previously [30]. The primers listed in Table 1 were used to amplify PCR products from plasmids containing the target sequences. The insert was ligated to the vector pET32c, and the plasmid was transformed into the BL21 competent cell (Invitrogen) for protein production. Positive colonies identified by white-blue selection were selected and confirmed by PCR and sequencing. A colony with the correct sequence from each was selected and used further to produce the recombinant protein.

**Table 1:** Primers used to produce recombinant proteins, LsPxtl-1 and LsPxtl-2. The restriction sites are indicated by underlined letters.

Primer	Forward (*Bam*H I restriction site)	Reverse (*Hin*d III restriction site)
LsPxtl-1	ACGCGGATCC**GA**atggtaagccaatggg	ACGCAAGCTT**AG**gaataaattaagattc
LsPxtl-2	ACGCGGATCC**GA**atgctgatgtttccca	ACGCAAGCTT**AG**tacttgtttcagtctt

### 2.4 Preparation of recombinant protein and whole sea lice crude protein vaccines

#### 2.4.1 Recombinant protein

To produce the recombinant vaccines, the clones containing LsPxtl-1 and -2 were incubated in LB broth containing 100 μg/ml Ampicillin overnight at 37 °C with continuous shaking. Next, protein purification was performed according to the protocol for the Overnight Express™ Autoinduction System 1 (Novagen, Merck KGaA, Darmstadt, Germany), followed by incubation at 37 °C overnight with continuous shaking. The cultures were then centrifuged, and the supernatant was removed. Pellets were washed with PBS and resuspended in PBS with a proteinase inhibitor. Next, the inclusion body fractions were sonicated (cycle .5, amplitude 60) for 40 min, including multiple breaks (1-2 min) to avoid overheating, and kept cool on an ice bath. The protein samples were then centrifuged, and the supernatant was removed. PBS with proteinase inhibitor was then added to the pellet.

**Fig. 1:**
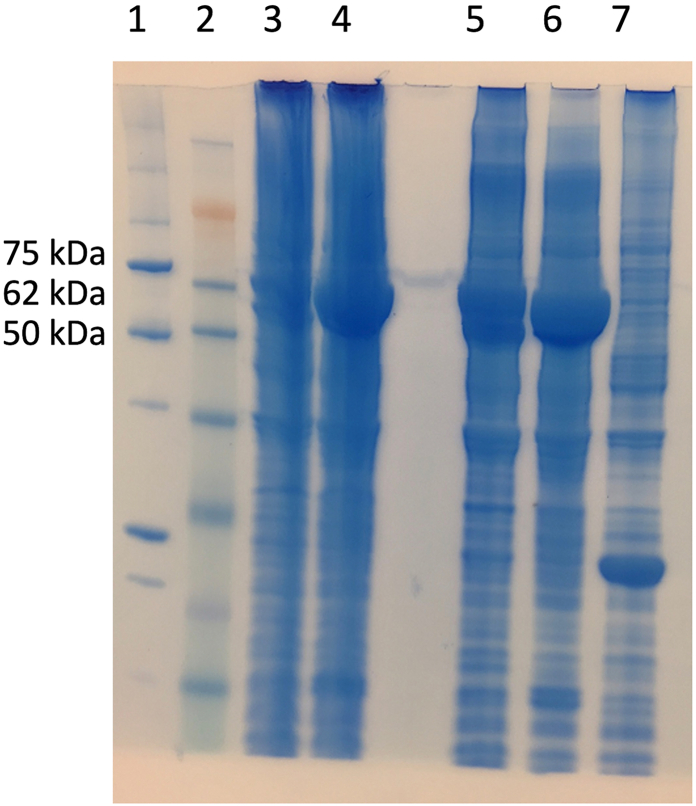
Validation of recombinant peroxinectin-like proteins with SDS-PAGE. 1. Precision Plus Protein™ All Blue Pre-stained Protein Standards. 2. SeeBlue™ Plus2 Pre-stained Protein Standard. 3 LsPxtl-1 inclusion body (IB), 20 min sonication, 4. LsPxtl-2 IB, 20 min sonication. 5. LsPxtl-1 IB, 40 min sonication. 6. LsPxtl-2 IB, 40 min sonication. 7 pET32c vector without insert (irrelevant protein). LsPxtl-1 had two bands, the first at 62 kDa (kDa) the second at around 58 kDa. Protein weight for LsPxtl-2 was about 75 kDa.

The concentrations of the inclusion body fractions and the whole sea lice crude protein were measured by Bio-Rad protein assay (Bio-Rad, California, USA) with a standard dilution using bovine serum albumin. Readings were performed using a Tecan Genios spectrophotometer (Tecan, Männedorf, Switzerland) at 595 nm. The expression of the recombinant proteins with the correct size was verified by SDS-PAGE using GelCode Blue Safe Protein Stain (Thermo Fisher Scientific). LsPxtl-1 consisted of two bands at around 62 and 58 kDa, while LsPxtl-2 had a weight of around 75 kDa Fig. 1. The adjuvant Montanide ISA 763 A VG (Seppic, Paris, France) was mixed with antigen at a ratio of 7.4 mL: 2.6 mL to form a water-in-oil emulsion vaccine.

#### 2.4.2 Whole sea lice protein

Whole sea lice crude protein (WSL) was prepared using salmon lice of both genders and various development stages, except for chalimus. The lice were collected from a non-medicinal delousing operation and immediately preserved in 10 % phosphate-buffered formalin. Antigen preparation was performed by washing the lice three times in PBS and storing them in 5 M NaCl overnight. PBS with a protease inhibitor was added to the lice to prevent protein degradation, and the lice were homogenized using a FastPrep-24 (MP Biomedicals, California, USA). After centrifugation to remove debris, the crude protein was resuspended in PBS and further sonicated (cycle 0,5, amplitude 50 for 40 min, including multiple breaks lasting 1-2 min to prevent protein from overheating). For the recombinant protein vaccines, water-in-oil emulsion vaccine preparations were prepared by mixing the adjuvant Montanide ISA 763 A VG with the WSL protein in a 7.4:2.6 (v/v) ratio.

### 2.5 Immunization trial and sampling

**Fig. 2:**
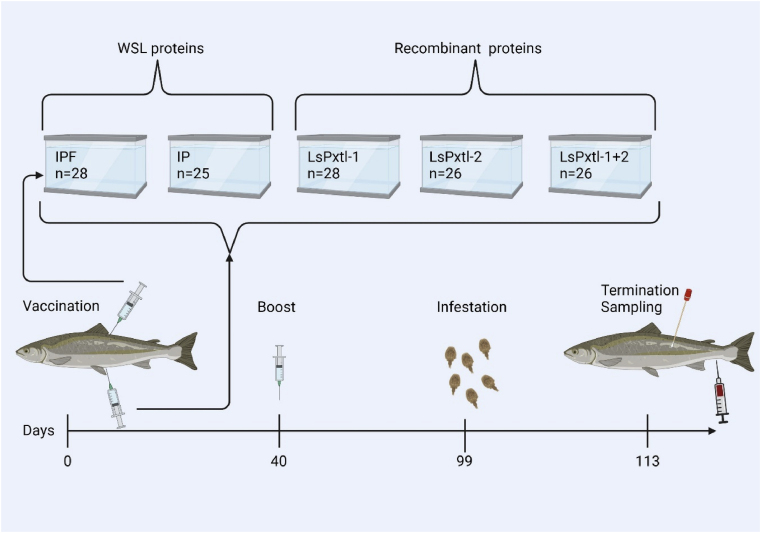
The timeline illustrates different timepoints of the immunization trial, administration method for each vaccine group and sampling material at end of immunization and challenge trial. Created with BioRender.com.

Five groups of Atlantic salmon, with an average weight of 81,6 g, were vaccinated intraperitoneally (ip) with the different vaccines listed in Table 2. Additionally, one group (IP–F) received an injection subcutaneously (sc) at the base of the dorsal fin. The details of the vaccination modalities, including the volume and amount (μg of protein), are outlined in Table 2. All groups were boosted 40 days (480°-days) post-primary immunization. Fish were kept in fresh water until they underwent smoltification. Smoltification was induced by exposing the fish to 24 h of light at 600°-days after vaccination and then transferring to seawater at 972°-days. All fish were kept together in one circular tank (3 m in diameter) with a water temperature of 12 °C. During the challenge, water flow was stopped for 30 min, and oxygen was added, after which water flow was resumed. Infestation with salmon lice (60 copepodites/fish) was carried out at 98 days (1176°-days) after vaccination. The trial was terminated, and fish were sampled 14 days (1344°-days) after the challenge when the lice had reached the pre-adult stage. Blood was collected in a Vacutainer (BD), and the plasma was separated from the red blood cells by centrifugation at 2500 rpm for 10 min. Sampling of mucus was performed using swabs according to the protocol for the Teco mucus collection set (TECO Medical, Sissach, Switzerland). An overview and timeline of the different vaccine groups and immunization time points are shown in Table 2 and Fig. 2.

**Table 2:** Overview of vaccine groups, type of vaccine, boosting, injection route, dosage given to the salmon, and number of fish used in the experiment.

Groups	Type	Administration route	Dose	Boost dose	No. of fish (total)	Designation
LsPxtl-1	Recombinant protein	IP	100 μg/0,1 ml	100 μg/0,1 ml	28	LsPxtl-1
LsPxtl-2	Recombinant protein	IP	100 μg/0,1 ml	100 μg/0,1 ml	26	LsPxtl-2
LsPxtl-1+2	Recombinant protein	IP	100 μg/0,1 ml	100 μg/0,1 ml	26	LsPxtl-1+2
Pet-vector	Recombinant protein without inserts	IP	100 μg/0,1 ml	100 μg/0,1 ml	28	PET
IP	Whole sea lice crude protein	IP	30 μg/0.1 ml	30 μg/0.1 ml	25	IP
IP + fin (F)	Whole sea lice crude protein	IP + fin injection	30 μg/0.1 ml F: 10 μg/0,05 ml	30 μg/0,1 ml F: 10 μg/0,05 ml	28	IP-F
PBS	PBS	IP	0,1 ml	0,1 ml	23	PBS
PBS + fin (F)	PBS	IP + fin injection	0,1 ml F: 0,05 ml	0,1 ml F: 0,05 ml	28	PBS-F

### 2.6 Custom-made anti-IgT

**Fig. S1:**
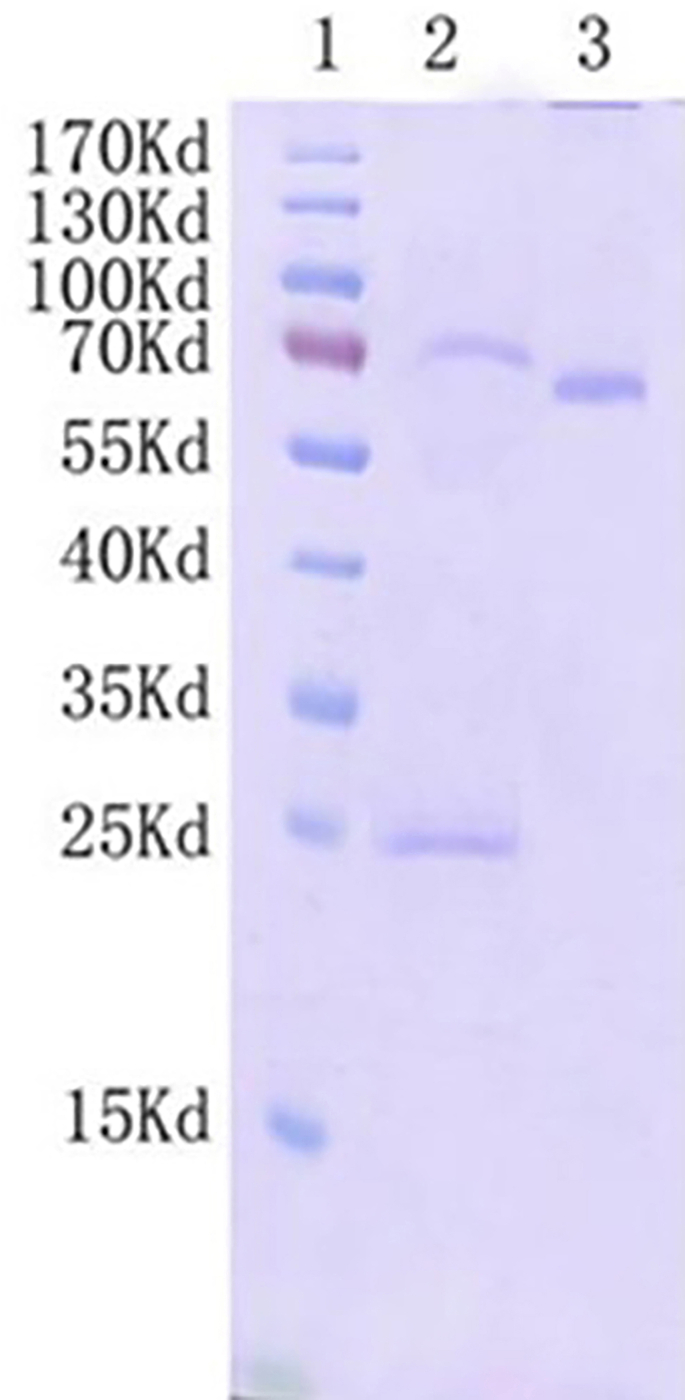
SDS-Page of IgT (lane 2, reducing conditions) showing heavy chain (≈75 kD) and light chain at 25 kD.

The anti-IgT antibody used to measure IgT in plasma and mucus was produced by Hangzhou HuaAn Biotechnology Co., Ltd, China. Two healthy New Zealand white rabbits were immunized subcutaneously with purified salmon IgT. The rabbits were boosted three times before plasma was collected, and anti-salmon IgT was purified using the IgT affinity column (Protein A/G/L affinity column). The molecular weight of the purified IgT was identified through SDS-PAGE, estimated to be 75 kDa for the heavy chain and 25 kDa for the light chain, respectively (Supplementary Fig. S1).

### 2.7 Validation of ig (IgM/IgT) and IgT antibodies

A Western blot analysis was performed to validate the antibodies. Albumin depletion was performed prior to Western blot using Pierce™ Albumin Serum Depletion Kits (Thermo Scientific), following the manufacturer's protocol. For the reduced samples, 30 μL of purified and diluted plasma (1:20 in PBS) from an Atlantic salmon was mixed with 15 μL of 4x Laemmli Sample Buffer (Bio-Rad) and NuPAGE sample reducing agent (Invitrogen). The mixture was heated at 70 °C for 10 min before being placed on ice. When running a non-reduced sample, the reducing agent was replaced with the sample, and no heating was applied before it was placed in the gel. The ladders (Precision Plus protein All Blue Prestained Protein Standard; 5 μL (Bio-Rad), MagicMark XP Western Protein Standard; 5 μL (Invitrogen) and the premixed and heated (reduced) and native (non-reduced) sample were loaded (15 μL) into a 5-12% Mini PROTEAN TGX stain-free protein gel (Bio-Rad) placed in a Mini-PROTEAN Tetra Cell filled with 1x Tris/Glycine/SDS electrophoresis buffer. The voltage was set to 210 V, and the gel ran for 45 min. The proteins were transferred to a PVDF membrane using Trans-Blot Turbo Mini .2 μm PVDF Transfer pack and Trans-Blot Turbo Transfer System (Bio-Rad). The transfer was carried out under a constant voltage of 20V for 7 min. The membrane was blocked for 5 min in Everyblot Blocking buffer (Bio-Rad). Antibody was diluted; 1:2000 Mouse anti-salmonid HRP (Immunoprecise, 1 mg/ml, Victoria, Canada) and 1:10 000 custom-made rabbit anti-IgT in full strength every blot blocking buffer for 30 min on a shaker at room temperature. Membranes were rinsed five times for 5 min in TBST (1x Tris-buffered saline with Tween 20) while shaken. Donkey anti-rabbit HRP (Cytiva, Amersham, United Kingdom) diluted 1:2000 was added to rabbit anti-IgT-probed membranes. Membranes were rinsed six times for 5 min with TBST. Clarity Western ECL substrate (Bio-Rad) was applied to the membranes and incubated for 5 min before using the ChemiDoc MP imaging system (Bio-Rad) to capture the chemiluminescent signal. The result was analyzed using the Image Lab software (Bio-Rad).

### 2.8 Enzyme-linked immunosorbent assay (ELISA) and validation

Salmon lice (*L. salmonis*) from different life stages, preserved in 10% formalin (copepodites, preadults, and adults of both genders), were washed three times in PBS. A protease inhibitor in PBS was added, and lice were homogenized using FastPrep-24 (MP Biomedicals). The homogenate was cleared by centrifugation, and the protein concentration in the supernatant was determined (0,44 mg/ml) before being aliquoted into small portions kept at – 80 ֯ C. A protein free extraction buffer (provided by the TECO Mucus Collection Set) was added to the swab containing the collected mucus following the instruction manual supplied by the TECO Mucus Collection Set (TECO Medical). Prepared samples were stored at −20 °C.

#### 2.8.1 Specific Ig(IgM/IgT) and IgT

The ELISA plates (Thermo Fisher Scientific-Nunclon) were coated with lice homogenates diluted 1:1000 in bicarbonate buffer (.795 g Na_2_CO_3_ + 1.465 g NaHCO_3_ + dH_2_O, up to 500 ml) and incubated at 4 °C overnight. The plates were washed three times with 250 μL/well of washing buffer (PBS/T) using a Biochrom Asys Atlantis automated 96-plate washing machine (Biochrom, Cambridge, United Kingdom). Next, 250 μL/well of blocking buffer (5% fat-free dry milk (Bio-Rad) in PBS+0,05% Tween (PBST)) was added, and the plates were incubated for 2 h at RT. The plates were washed three times, as above, and 100 μL of diluted salmon plasma (1:400 in diluent buffer, 1% fat-free dry milk in PBST) or 50 μL of mucus mixed with extraction buffer diluted 1/10 in the diluent buffer was added. The plates were then incubated at 4 °C overnight.

After overnight incubation, the plates were washed as described above before the addition of antibodies. For specific IgM/IgT, 100 μL of HRP-labeled antibody (1 mg/mL) of mouse anti-salmonid immunoglobulin (Immunoprecise) diluted 1:2000 was added and incubated for 1 h at RT. The plates were rewashed before 75 μL of TMB substrate solution (Abcam, Cambridge, United Kingdom) was added and incubated for 5 min in the dark at RT. The reactions were stopped with 50 μL of 1 M HCl. Finally, Tecan Genios and Tecan Spark spectrophotometers (Tecan, Männedorf, Switzerland) were used to measure absorbance at 450 nm.

For the specific IgT, on the other hand, a custom-made anti-IgT salmonid antibody prepared in rabbits was used at a dilution of 1:10 000 and incubated for 1 h. Plates were washed five times, and then donkey-*anti*-rabbit HRP was added and incubated for 1 h to prepare for detection. The plates were washed 5 times, and 75 μL of TMB substrate solution was added and incubated for 5 min in the dark at RT. The reactions were stopped with 50 μL of 1 M HCl. Finally, Tecan Genios and Tecan Spark spectrophotometer (Tecan, Männedorf, Switzerland) were used to measure absorbance at 450 nm.

### 2.9 Statistical analysis

Statistical analyses were conducted in Stata (v18; StataCorp LLC, USA). Group differences in fish weight/length were assessed using the Kruskal–Wallis test followed by Dunn's multiple comparisons. Lice counts were analyzed using negative binomial regression due to overdispersion; model outputs are reported as effect estimates with 95% confidence intervals and associated p-values. Data handling and visualization were performed in Python (v3.14.2; packages e.g., numpy, pandas, scipy, matplotlib, seaborn), including kernel density estimates and violin/boxplot visualizations. Chord diagrams were generated in R (2025.09.1; package e.g., circlize). Where applicable (e.g., Fig. 13), Pearson correlations were computed separately within lice-burden strata and bootstrap resampling (n = 1000 iterations) was used to derive 95% confidence intervals.

## 3 Results

### 3.1 Vaccination and challenge trial

The recombinant and whole sea lice antigen preparations were used in a vaccination and challenge trial to evaluate their ability to elicit a systemic and local antibody response (in mucus) and to reduce lice infestation. The different groups consisted of 23-28 fish: IP (n = 25), PBS (n = 23), IP-F (n = 28), PBS-F (n = 28), LsPxtl-1 (n = 28), LsPxtl-2 (n = 26), LsPxtl-1+2 (n = 26), and PET (n = 28). Fifteen fish from each group were randomly selected for analysis of antibody responses. Statistical analysis revealed no significant differences in weight and length between the different groups and the control group, PBS, except for LsPxtl-2, where fish were found to be significantly lower in weight (p = 0.0078) and length (p = 0.0072).

### 3.2 Validation of antibodies

**Fig. 3:**
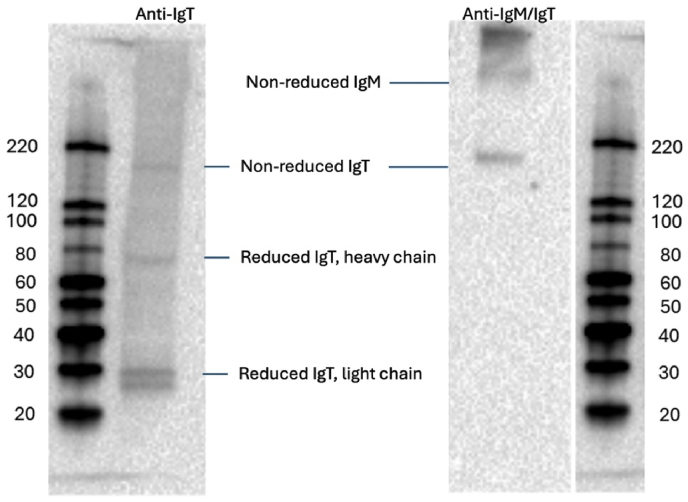
Western blot results validated rabbit anti-salmon IgT (IgT) specific for IgT, while mouse anti-salmonid Ig detected both IgM and IgT in salmon plasma. Reduced samples were used for rabbit anti-IgT, stained heavy and light chains of IgT, at 75 kDa and 25-30 kDa. In non-reduced samples used for mouse anti-Ig, IgM was more than 220 kDa, while IgT was around 180 kDa.

Before studying the antibody response, the secondary antibodies were tested for their reactivity to plasma immunoglobulins (IgM and IgT) using a Western blot (Fig. 3).

The custom-made rabbit anti-IgT, reacted positively for bands at 75 (heavy chain), 25-27 (light chain), and 180 kDa, the same weight as the non-reduced IgT protein. The mouse anti-salmonid Ig (conjugated with HRP) stained bands at >220 kDa (IgM) and at 180 kDa (IgT), but no staining was achieved for reduced samples.

### 3.3 Lice numbers

**Fig. 4:**
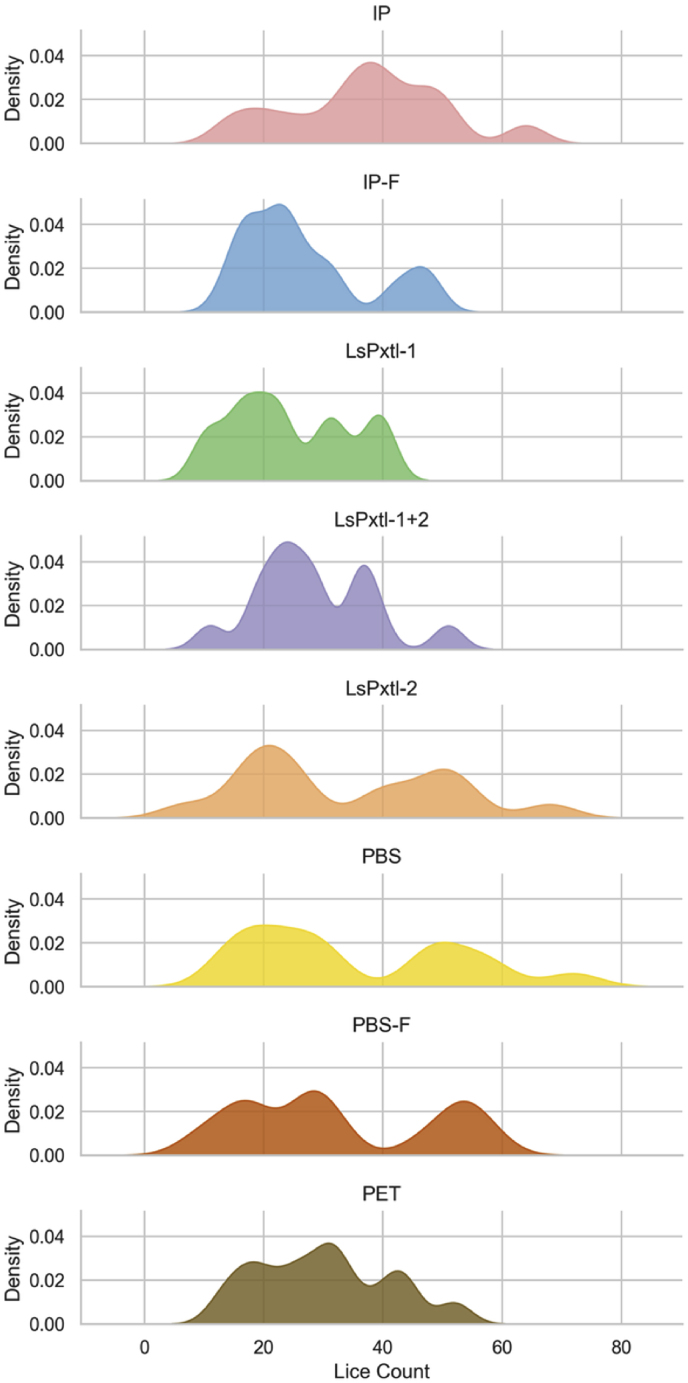
Kernel density plot for lice counts along the x-axis and distribution on the y-axis. All groups showed a multimodal dispersion.

Salmon lice were collected at pre-adult stage for optimal evaluation of protection. The lice numbers in the different groups (vaccinated and controls for the selected 15 fish/group) are summarized in Table 3. The median deviates from the mean values, indicating a degree of skewness in the lice numbers. This was further analyzed using kernel density plots, which showed multimodality for almost all groups (Fig. 4).

**Table 3:** Summary of mean, standard deviation (SD), median, interquartile range (IQR), min, and max values for lice numbers in the different vaccine groups at the time of counting.

Vaccine groups	Mean	SD	Median	IQR	Min	Max	N
IP	37.2	13.1	38	20	14	64	15
IP-F	26.9	10.6	24	15	14	48	15
LsPxtl-1	24.6	10.0	22	17	10	40	15
LsPxtl-1+2	28.5	9.8	27	14	11	51	15
LsPxtl-2	33.7	17.2	25	31	7	68	15
PBS	35.5	18.0	30	29	14	72	15
PBS-F	32.3	16.3	28	35	9	58	15
PET	30.1	11.1	31	20	13	52	15

**Fig. 5:**
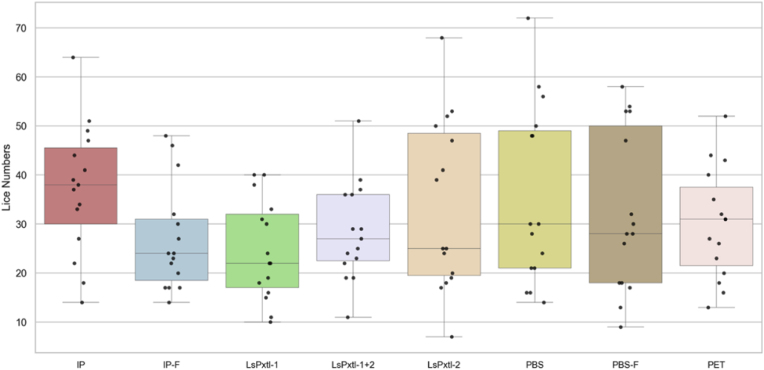
Lice numbers in the different vaccine groups. Lice numbers in the different vaccine groups and controls, showing the 25/75% quantile, median, and 95% CI. Each dot represents the number of lice in an individual fish.

The multimodality observed in Fig. 4 is further supported by box plots, which show a separation into subgroups of low and high lice numbers for many of the groups, particularly LsPxtl-1, LsPxtl-2, PBS, and PBS-F (Fig. 5).

As a result of over-dispersed lice count data, negative binomial regression was used to assess statistical significance. The lice numbers for the different vaccines were compared to corresponding controls, including PBS, PBS + F, and a PET (irrelevant protein) control, as summarized in Table 4. The LsPxtl-1 vaccine performed best, with vaccinated fish showing significantly lower lice numbers than PBS controls (p < 0.05). Against the IP-PET (irrelevant recombinant protein), the difference was not significant (Table 4). The IP-F group was the second best, with a trend toward lower lice numbers compared with PBS group (Table 4) at p < 0.1 (p = 0.074), while no difference was observed when tested against PBS + F (p = 0.243). The remaining vaccine groups did not differ significantly from the controls.

**Table 4:** Comparison of different vaccine groups regarding the number of lice obtained when compared to various control groups, as indicated. The p-values for negative binomial regression analysis are given in each cell.

Groups	Type	Administration route	Compared to PBS (IP), p-values	Compared to PBS + F, p-values	Compared to irrelevant protein (PET), p-values
LsPxtl-1	Recombinant protein	IP	.029∗	.112	.157
LsPxtl-2	Recombinant protein	IP	.780	.823	.496
LsPxtl-1+2	Recombinant protein	IP	.161	.433	.673
IP	Whole sea lice crude protein	IP	.764	.381	.112
IP-F	Whole sea lice crude protein	IP + fin injection	.085	.266	.406

∗p < 0.05.

The mean antibody levels in plasma and mucus and the differences versus PBS for the WSL groups IP and IP-F and the recombinant groups LsPxtl-1, LsPxtl-1+2, and LsPxtl-2 are shown in Supplementary Table 1.

**Fig. S2:**
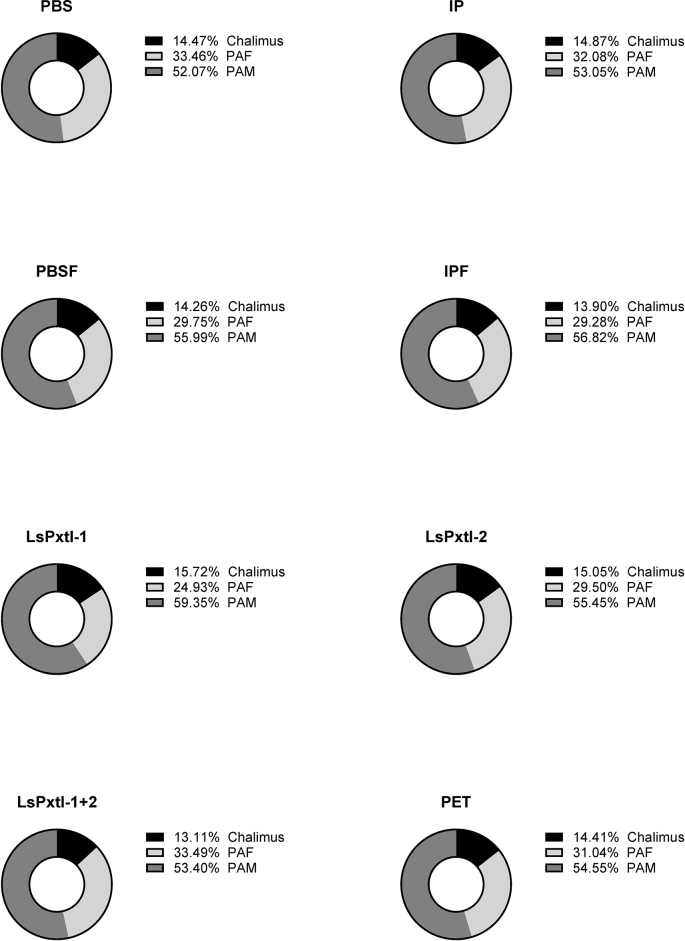
Sex distribution and developmentalstages of salmon lice for the vaccinated groups and control groups.

There were no notable differences in sex distribution or developmental stages of salmon lice between the vaccinated groups and controls (Supplementary Fig. S2). However, there was a higher proportion of males than females in the pre-adult stage across both vaccinated and control groups. The recombinant vaccine groups, LsPxtl-1 and LsPxtl-2, had more lice at the chalimus stage. Additionally, a higher percentage of pre-adult males was observed in the LsPxtl-1 group than in other groups (Supplementary Fig. S2).

### 3.4 Local and systemic antibody responses after vaccination

#### 3.4.1 Lice-specific plasma antibodies

**Fig. 6:**
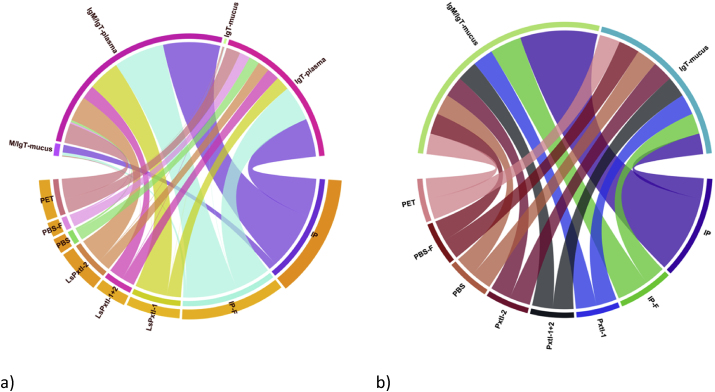
a,b. a) Chord plot with interconnections between the antibodies measured in different vaccine groups and controls, including average relative lice numbers, given as bars heights of vaccines/controls. Vaccines IP, IP-F, LsPxtl-1, and -2 have the highest IgM/IgT antibody levels in plasma, while IP and IP-F dominate for plasma IgT. b) IgT/IgM and IgT in mucus presented separately since interconnections are complex to ascertain in a). IP and IP-F have raised higher levels of IgM/IgT antibodies than other groups, even between groups for IgT.

Several parameters are included, and there are complex interactions that make it challenging to obtain an overview of each one, their relationships, and interactions for the different vaccine groups/controls, as well as the lice numbers. We prepared chord plots to provide an initial overview (Fig. 6). Here, the measured parameters are shown in the upper half, the vaccines/controls in the lower, and the relative lice numbers are shown below each vaccine/control group. IgM/IgT mean OD values, measured in plasma, are generally higher than those of IgT in plasma, and vaccines IP and IP-F show the highest levels for both. The recombinant vaccines are almost equal for IgT/plasma, while LsPtx-1 and LsPtxl-2 are highest for IgM/IgT for the recombinant vaccines (Fig. 6). IgT and IgM/IgT in mucus are very low, with IgT being the lowest. IP-F and LsPtxl-1 have the lowest bars/lice numbers.

**Fig. S3:**
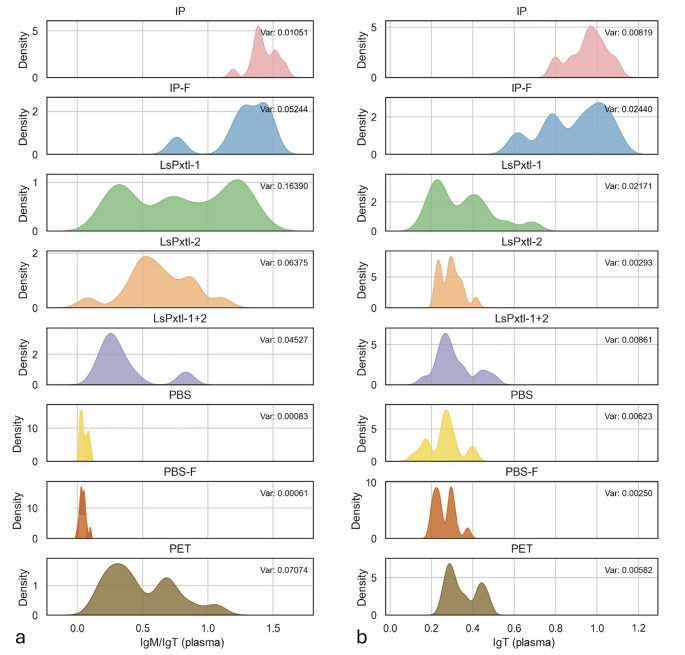
a) Kernel density plot for IgM/IgT (plasma). Distinct multimodal distribution for the vaccine groups, lesser for PBS and PBS-F. b) Kernel density plot for IgT (plasma). Distinct multimodality for vaccinated and control groups.

**Fig. S4:**
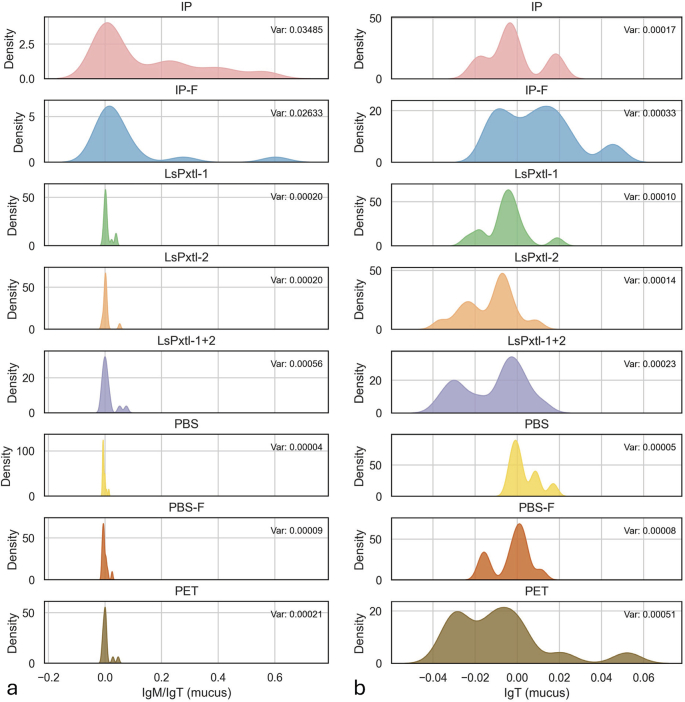
a) Kernel density plot for IgM/IgT in mucus found the IP and IP-F groups with widest spread and strongest right skew, with long upper tails. The other groups were narrowly distributed around very low values, although small secondary peaks were present in several groups. b) Kernel density plot for IgT in mucus showing a multimodal distribution for all groups.

The measured antibody levels for IgM/IgT and IgT in plasma and mucus are summarized in Table 5. Kernel density plots for each vaccine group and controls for lice-specific plasma IgM/IgT (Supplementary Fig. S3a) and plasma IgT (Supplementary Fig. S3b) and mucus IgM/IgT (Supplementary Fig. S4a) and mucus IgT (Supplementary Fig. S4b) are provided. The distribution of plasma antibodies varies between vaccine groups, with multimodal distributions for IgM/IgT in IP-F, LsPxtl-1, LsPxtl-1+2, and PET (Supplementary Fig S3a). In contrast, the most prominent multimodality is observed for plasma IgT in PBS-F and PET (Supplementary Fig. S3b).

**Table 5:** Mean OD_450_ values for IgM/IgT or IgT (only) in mucus and plasma are shown. The mean IgT in mucus is shown as values below zero for most groups since the obtained values were normalized against PBS.

Groups	IgM/IgT	IgT	IgM/IgT	IgT
	Mucus (mean)	Mucus (mean)	Plasma (mean)	Plasma (mean)
IP	.143	−.002	1.427	.956
IP-F	.078	.009	1.269	.888
LsPxtl-1	.007	−.005	.796	.349
LsPxtl-1+2	.009	−.011	.347	.31
LsPxtl-2	.004	−.013	.621	.292
PBS	−.003	.003	.051	.261
PBS-F	−.002	−.003	.041	.263
PET	.003	−.008	.495	.351

**Fig. 7:**
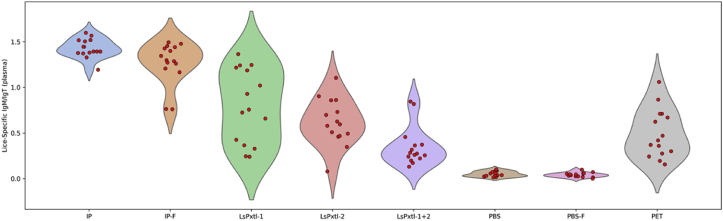
Violin plots for lice-specific plasma IgM/IgT responses for the different groups, tested against whole-lice antigens as a coat in the ELISA plates. Values for the y-axis are OD_450_.

**Fig. 8:**
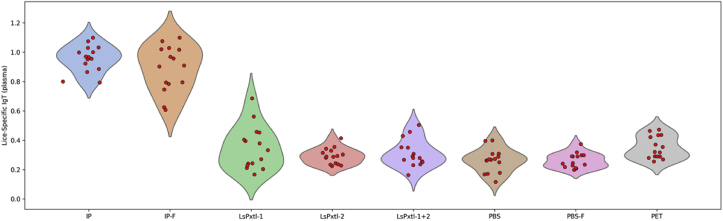
Violin plots for lice-specific plasma IgT responses for the different groups, tested against whole-lice antigens as coat in the ELISA plates. Values for the y-axis are OD_450_.

We then dived into the details of the lice-specific plasma IgM/IgT (Fig. 7) and IgT (Fig. 8), presented as violin plots with swarm overlay, for better illustration of the distribution within each vaccine group/control groups. The lower “tail” for lice-specific plasma IgM/IgT for the IP-F group is explained by two fish having low values, while the LsPxtl-1 and LsPxtl-2 OD-values show a wide distribution, and the PET control (Fig. 7). All vaccinated groups are significantly different from PBS/PBS-F and PET (p < 0.05), while LsPtxl-2 and LsPtxl-1+2 are not significantly different from PET (p > 0.05).

Lice-specific plasma IgT levels (Fig. 8) showed a wide distribution for LsPxtl-1 and IP-F (evenly distributed), while the other groups had less variation. Only IP and IP-F have antibody levels that are significantly higher than those of all control groups (p < 0.05). At the same time, the recombinant vaccines do not differ from any of the controls. All fish have measurable plasma IgT levels (Fig. 8).

#### 3.4.2 Lice-specific antibodies in mucus

**Fig. 9:**
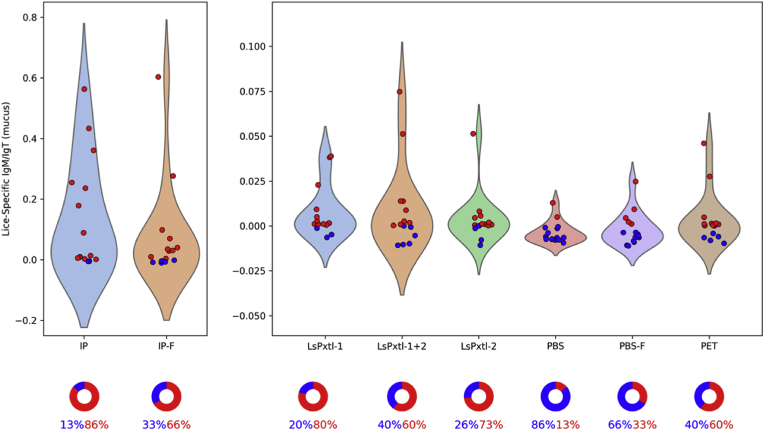
Violin plots for mucus lice-specific IgM/IgT responses for the different groups tested against whole-lice antigens as coats in the ELISA plates. Values along the y-axis are OD_450_. Blue dots are non-responders, and red dots represent responders. The distribution (%) of non-responders (blue)/responders (red) is shown in the pie charts below, along with violin plots.

**Fig. 10:**
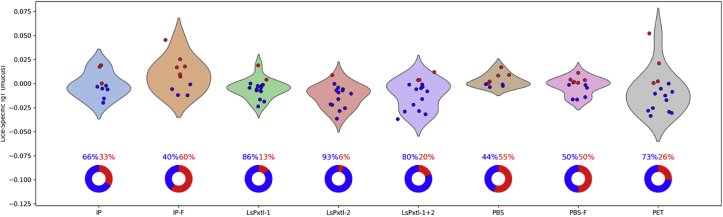
Violin plots for mucus lice-specific IgT responses for the different groups tested against whole-lice antigens as coats in the ELISA plates. Values along the y-axis are OD_450_. Red dots are positive values (responders), and blue dots represent values below the normalized reference (non-responders). The distribution (%) of non-responders (blue)/responders (red) is shown in pie charts below violin plots.

We found that lice-specific mucus IgM/IgT or IgT levels were low relative to those measured in plasma. The distributions of each are presented in Fig. 9, Fig. 10, respectively, and the values are listed in Table 5. Most groups have a high proportion of non-responders for IgM/IgT and IgT in mucus. The negative values, particularly for the IgT, result from normalization (non-vaccinated controls and non-challenged controls). Since a certain proportion in each vaccine group did not have measurable antibody levels, a pie chart is included to show the fraction of responders/non-responders. Fish vaccinated with WSL preparations (IP and IP-F) and recombinant vaccines LsPxtl-1 and LsPxtl-2 have mucus IgM/IgT levels significantly different (p < 0.05) from PBS; otherwise not. The fraction of responders versus non-responders varies between vaccine groups and controls (Fig. 9). For IP and IP-F-vaccinated fish, 86% and 66%, respectively, have measurable IgM/IgT antibodies. For the recombinant vaccines, 60–80% of the fish in these groups are responders, whereas in the two PBS groups, only 13.3% and 33.3% are responders (Fig. 9). The PET group stands out, with 60% of the fish being responders.

For lice-specific mucus IgT (Fig. 10), measurable levels are low, the IP group has 33% responders, and 60% for IP-F. Fish vaccinated with recombinant vaccines are, for the most part, non-responders; the highest fraction of responders, 20%, was found in LsPtxl-1+2 (Fig. 10). Fish in the PBS and PBS-F groups have 44.4% and 50% response rates, respectively. Statistically, the IP and IP-F groups have significantly higher levels than the controls (p < 0.05), whereas the recombinant vaccine groups do not (p > 0.05).

**Fig. 11:**
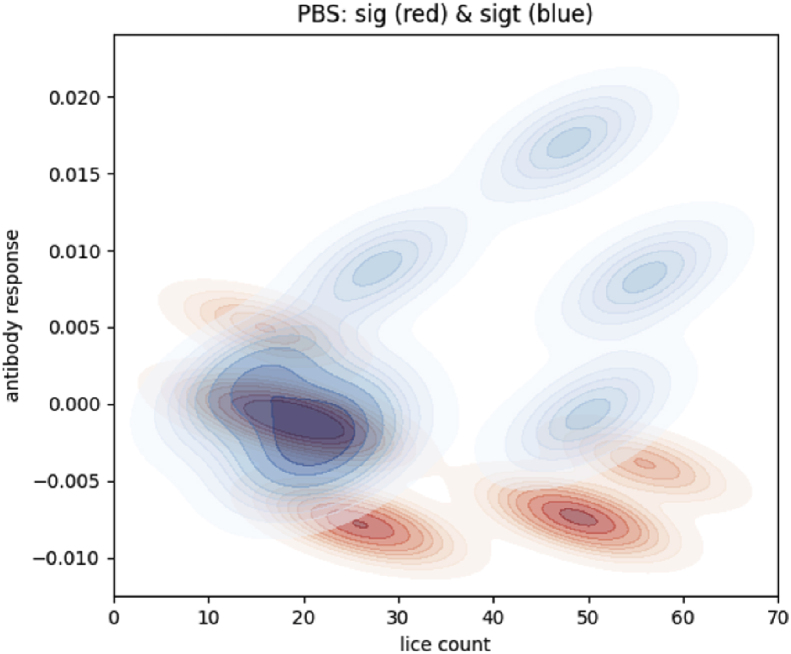
Two-dimensional kernel density plot for lice-specific mucus antibodies for the PBS group. IgM/IgT (sig, red color) and IgT (blue color) plotted against lice numbers per fish (x-axis).

There are no differences between the IP and IP-F groups and controls (PBS) in terms of the number of fish being responders (p > 0.05). For the recombinant vaccines, there is a statistically higher number of responders in the PBS or PBS-F controls compared to LsPxtl-1 (p < 0.0001), LsPxtl-2 (p < 0.0001), and LsPxtl-1+2 (p < 0.0001). Compared to the PET control, LsPxtl-1+2 is not significantly different (p = 0.214); otherwise, it is. If this is a response to the infection per se, in the PBS group, it is challenging to elucidate. We prepared a 2D-KDE plot for lice-specific IgM/IgT (sig) and IgT (sigt) versus lice numbers (Fig. 11), and there is a distinct trend that IgM/IgT levels decline while IgT levels increase. The trends for the IP-F and PET controls differed.

#### 3.4.3 Relationship between measured anti-lice antibody responses and lice numbers

We then plotted the number of lice against antibody levels in the vaccinated and control groups. There was no correlation between lice-specific IgM/IgT or IgT in plasma or mucus, evident from the scatterplots (Supplementary Fig. S5a and S5b). Negative binomial regression analysis (used due to overdispersion of data) for the same gave no significant results for either (p > 0.05). Similarly, no significant association was detected between lice burden and lice-specific IgM/IgT or IgT measured in mucus (negative binomial regression; p > 0.05).

#### 3.4.4 Antibody levels by low-high lice numbers

The lice number of lice by vaccine group showed a relatively broad distribution (high variation) for almost all vaccines (Fig. 5). Within most groups, there is a separation into high and low lice numbers (Fig. 5). Such variation can mask underlying correlations between variables, e.g., antibody levels and lice numbers. On this basis, we examined differences in lice-specific Ig levels for subgroups of fish with high (>25) or low (<25) lice numbers. The cut-off of 25 lice/fish was based on the average lice count observed in the LsPtxl-1 group, the best-performing vaccine (Table 3). First, we compared the mucus and plasma IgM/IgT responses for the groups vaccinated with whole lice preparations, IP and IP-F, and separated into the two lice burdens (Fig. 12a, Fig. 12b). There was no clear relationship between antibody levels and lice burden.

**Fig. 12a:**
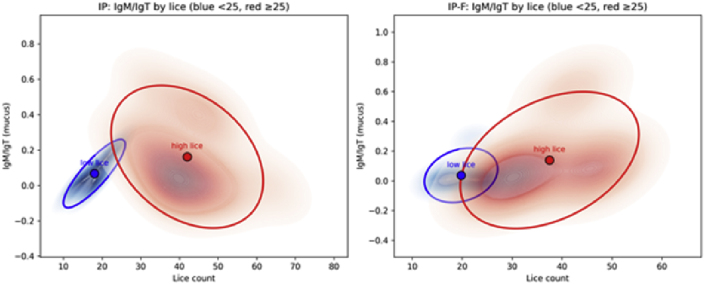
2D kernel density estimates (KDEs) with centroids and 95% confidence ellipses to visualize IgM/IgT in mucus and lice distributions for different lice burden, <25/fish and ≥25 lice/fish.

**Fig. 12b:**
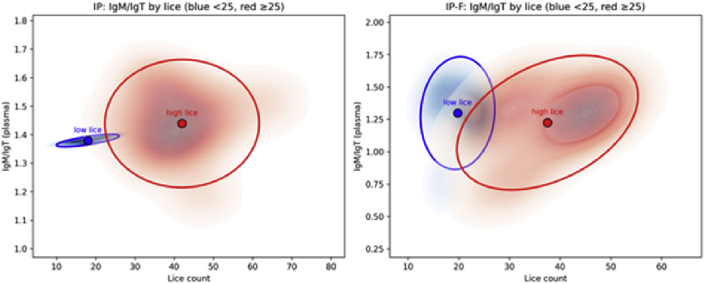
2D kernel density estimates (KDEs) with centroids and 95% confidence ellipses to visualize IgM/IgT in plasma and lice distributions for different lice burden, <25/fish and ≥25 lice/fish.

**Fig. 13:**
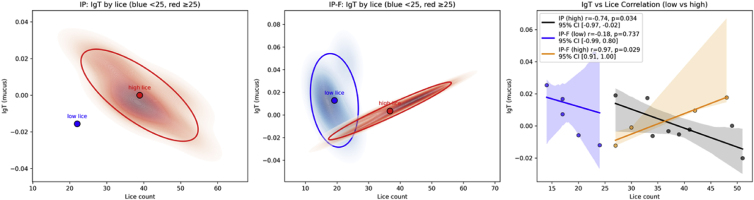
2D kernel density estimates (KDEs) with centroids and 95% confidence ellipses to visualize IgT in mucus and lice distributions. These represent a stratified correlation analysis using Pearson's r to assess IgT–lice relationships, separately for low and high lice groups, and include bootstrap resampling (1000 iterations) to compute 95% confidence intervals for the correlation coefficients.

The same trend was observed for IgT in plasma, while for IgT in mucus, an interesting trend emerged (Fig. 13). First, the IP vaccinated fish has a higher lice number than the IP-F group, 36.2 vs. 26.9 (p = 0.001), lower IgT in mucus, and a negative correlation between lice number and IgT for the high lice burden group (Fig. 13). For IP-F, IgT at low lice burden is higher than at high burden, and interestingly, fish with high lice burden show a positive correlation between IgT levels and increasing lice numbers (Fig. 13), and it is tempting to speculate if the infestation itself increases the IgT response in mucus.

## 4 Discussions

The study demonstrates that antigen source and vaccination modality influence the immune response and effect on sea lice infestation in Atlantic salmon under experimental conditions. The recombinant LsPxtl-1 vaccine was given i.p., and whole lice antigen preparations were given as a combined i.p./s.c. administrations reduce lice counts after challenge. While a significant reduction in lice numbers was observed, lower lice numbers in general did not correlate with circulating or mucosal antibody levels, except for a minimal trend for mucus antibodies with the two best-performing vaccine preparations. It is likely that other immune mechanisms, not detailed here, are involved in protective responses against infestation.

Studies have suggested that specific IgT and other host factors, such as complement, may have a central role in protecting the fish against the Ich parasite in immunized fish skin [32]. Zhang et al. [16] reported increased levels of IgT ^+^ cells in the *lamina propria* and IgT in the gut mucus of salmonids that had survived infestation with the parasite *Ceratomyxa shasta*, compared to non-infected control fish. An upregulation of the gene encoding IgT was also observed, while no differences in concentration and upregulation were seen for IgM in surviving fish. Measured immune responses were asymmetric, with parasite-specific IgM detected in plasma and not in the gut. In contrast, parasite-specific IgT was detected in gut mucus but not in the plasma [16]. These studies were carried out in naïve fish, and compared to our study, the non-vaccinated groups were examined, apart from the specific parasite being studied. We found that 50-55% of the naïve PBS/PBS-F groups had measurable mucus IgT levels, 26% for the PET group, and the levels increased with higher lice numbers, indicating a response induced by the infestation.

We do not indicate an asymmetric immune response in naïve fish. It is important to note that recent studies have modified the dichotomous plasma/mucus response. IgT was found highly upregulated in gilthead seabream after infestation with the intestinal parasite *Enteromyxum leei* [33]. Mucosal vaccination with *Photobacterium damselae* subsp. *piscicidae* followed by pathogen challenge induced systemic IgM and local IgT responses [33] indicating a finely tuned local response following immunization and/or challenge.

The complexity of potential cross-talk between local and systemic responses, as well as previous studies [34] showing an impaired effect of combined parenteral and mucosal vaccination against sea lice infestation, motivated us to explore a combined i.p. and subcutaneous vaccination modality for vaccines based on whole lice preparations. The modality chosen was based on studies showing that fin tissue in teleosts (e.g., zebrafish) mounts a local immune response following intra-fin administration [23]. They are covered by the same mucosal epidermis as skin therefore part of SALT [25]. Additionally, salmon lice preferentially attach to the dorsal surfaces including fin regions [26]. Despite a significant reduction in lice numbers for the IP-F group compared to the IP group, we were unable to correlate this with systemic or local Ig responses directly. These findings suggest that humoral responses (systemic or local) do not play a crucial role in protecting against lice infestation. However, the specificity of the antibodies needs to be considered, which was not assessed in this study. It should be added that the number of fish included in the study (in the different groups) is limited. The fraction of responders (IgT) in the IP-F group is higher than in the IP group, although the difference is not statistically significant. Our findings align with previous studies, which have detected low levels of local and systemic antibodies in PBS-injected fish, suggesting that site-induced immunomodulation of host responses may play a role [35,36]. It is noteworthy, however, that a shift from higher IgM/T production to more IgT production was observed upon subcutaneous fin injection. Whether this shift contributes to the reduction in lice number observed per se or whether it reflects a change in the type of inflammatory response induced remains unknown. In any case, this finding suggests that subcutaneous injection results in modulation of local immune responses, which impacts lice infection. The nature of these modulations warrants further investigation. Finally, we did not include a group with subcutaneous fin injection-only, and we do not know whether this injection route alone can induce mucosal antibody responses, which should be investigated in future studies.

LsPxtl-1 had a significantly lower number of lice than the control (PBS). The heme peroxidase LsPxtl-1 in the salmon louse was tested for its function, location, and expression, and used as an antigen for vaccination against sea lice infestation [30]. It was found essential for development, as knockdown in the nauplius stage caused poor swimming performance and death after molting to copepodite. *In situ* hybridization revealed that the protein was localized in the ovaries, gut, and egg strings of adult female lice, indicating its importance during development and, thus, its potential as a vaccine candidate. However, the vaccine given as DNA resulted in only low protection and a slight (non-significant) reduction in the number. Several factors influence vaccine efficacy, including modality, boost protocols, antigen concentration, and immunogenicity. In the current study, the antigen was given as a protein, with a higher dosage and boosted 40 days post-immunization. While the use of whole lice antigens is of interest experimentally, recombinant antigens would be the choice from a vaccine development standpoint. The results obtained for LsPxtl-1 make it an interesting candidate antigen, and findings, compared with previous studies, suggest that better protection can be achieved by improving formulations and delivery methods. The mechanism by which this protection is conferred remains unclear. A tendency towards higher systemic IgM/T and IgT responses suggests that antibody responses may play a role in protection.

In contrast, the lack of correlation between antibody levels and the reduction in lice number indicates the opposite. Hence, the nature of the protective responses induced should be further investigated in future studies. One of the factors that could have contributed to the inconclusive results is that antibodies were measured at the end of the experiment, which makes it challenging to distinguish between vaccine-induced and infection-induced responses. A sampling right before infection time would have been helpful in this regard.

A limitation of this study is the method used to collect mucus. Although this method has been validated and commercialized for use in zebrafish, it is not optimized for use on Atlantic salmon. The low antibody levels in mucus are likely influenced by the suboptimal sampling technique, which uses small swabs and handling of fish prior to mucus collection, causing loss of mucus. Optimizing the collection method and handling fish less before sampling would possibly improve the detection of antibodies in the mucus. Due to the experimental setup, only 15 fish were randomly selected for sampling at the trial's termination to analyze responses in plasma and mucus in relation to lice numbers. Unfortunately, some mucus samples were lost during sample preparation and optimization of the ELISA, resulting in fewer individuals for the specific IgT measurement groups: IP-F, IP, PBS-F, and PBS. Another limitation was that the recombinant antigens were administered only by ip route and not by fin injection, in contrast to the WSL antigens. Due to the limited number of fish groups available, only the WSL antigens could be tested using both ip and ip + fin delivery.

The results from the Western blot show that the molecular weight of the antibodies used in the study is the same as that found in other studies [16]. Non-reduced samples exhibit a clear difference in molecular weight, with IgM (greater than 220 kDa) and IgT (180 kDa). Under reduced conditions, we find the heavy chain of IgT at 75 kDa and the light chain at 25-27 kDa, consistent with the results presented by Zhang et al. [16]. The findings also highlights the importance of validating the commercial antibodies against fish immunoglobulins before using them for detecting the specific isotypes, especially that knowledge are still developing regarding the roles and the presence of these different isotypes are still developing.

The sex distribution of the lice attached to the fish showed a higher proportion of males in all vaccine groups and controls. This observation is even more pronounced for the LsPxtl-1, which has more lice still in the chalimus stage. The immune response may target the developmental stage of the lice, thereby delaying the maturation of female lice; however, this remains to be further explored. The sex imbalance observed was also reported in a previous study [30] where both the vaccination and challenge trial, as well as the RNAi study for LsPxtl-2, showed an accumulation of male lice attached to the fish.

## 5 Conclusion

Earlier studies examining the role of IgT in fish focused on the expression levels of IgT/IgM and the detection of antibodies using western blotting. Few studies have investigated the relative levels of antibodies produced and secreted in different host compartments, such as the plasma and mucus. Overall, the results show that the reduction in lice numbers post-vaccination depends on the antigen and vaccination modality; however, additional optimization of the vaccines is needed to improve efficacy and to understand how protective immune mechanisms protect against lice infestation.

## Declaration of competing interest

The authors declare that they have no known competing financial interests or personal relationships that could have appeared to influence the work reported in this paper.

## Figures and Tables

**Fig. S5a:**
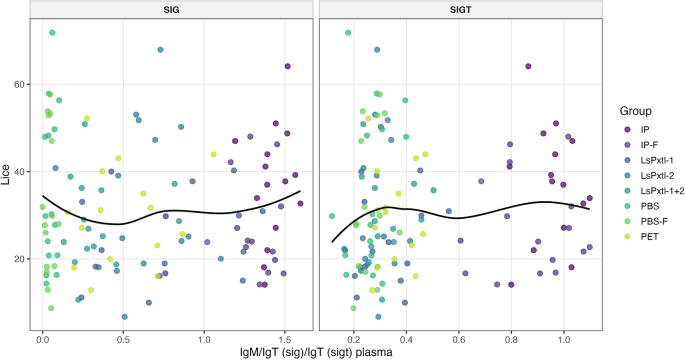
Scatterplot of lice numbers versus plasma OD values for IgM/IgT (SIG) and IgT (SIGT) across vaccine groups. Points are colored by group. There was no clear overall relationship between antibody levels and lice numbers.

**Fig. S5b:**
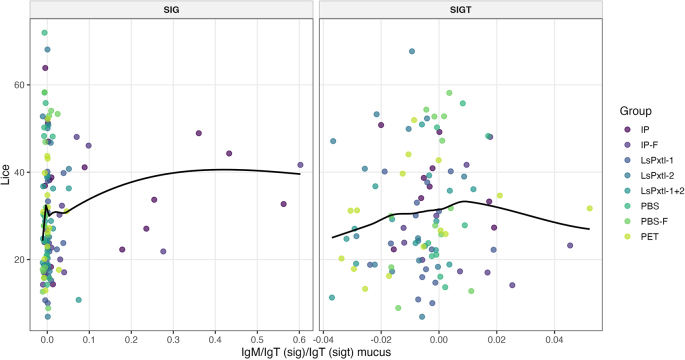
Scatterplots of lice numbers versus mucus antibody OD values for IgM/IgT reactive with SIG and IgT reactive with SIGT across vaccine groups. Points are colored by group. SIG-reactive mucus values were strongly clustered near zero, while SIGT-reactive values showed a broader spread, but neither panel showed a clear overall relationship with lice numbers.

## Data Availability

Data will be made available on request.

## References

[1] Ruggesæter A., Haug K.M.V. (2021).

[2] Aaen S.M., Helgesen K.O., Bakke M.J., Kaur K., Horsberg T.E. (2015). Drug resistance in sea lice: a threat to salmonid aquaculture. Trends Parasitol..

[3] Barrett L.T., Overton K., Stien L.H., Oppedal F., Dempster T. (2020). Effect of cleaner fish on sea lice in Norwegian salmon aquaculture: a national scale data analysis. Int. J. Parasitol..

[4] Groner M.L., Laurin E., Stormoen M., Sanchez J., Fast M.D., Revie C.W. (2019). Evaluating the potential for sea lice to evolve freshwater tolerance as a consequence of freshwater treatments in salmon aquaculture. Aquaculture Environment Interactions.

[5] Grøntvedt R.N., Nervikbø I., Viljugrein H., Lillehaug A., Nilsen H.G.A. (2015). Norwegian Veterinary Institute'S Report (Norwegian Veterinary Institute).

[6] Hannisdal R., Nøstbakken O., Hove H., Madsen L., Horsberg T., Lunestad B. (2020). Anti-sea lice agents in Norwegian aquaculture; surveillance, treatment trends and possible implications for food safety. Aquaculture.

[7] Myhre Jensen E., Horsberg T.E., Sevatdal S., Helgesen K.O. (2020). Trends in de-lousing of Norwegian farmed salmon from 2000–2019—Consumption of medicines, salmon louse resistance and non-medicinal control methods. PLoS One.

[8] Sommrset I.W.-N.J., Moldal T., Oliveira V.H.S., Svendsen J.C., Haukaas A., Brun E. (2024). Veterinærinstituttet Rapportserie Nr.

[9] Grayson T.H., Jenkins P.G., Wrathmell A.B., Harris J.E. (1991). Serum responses to the salmon louse, *Lepeophtheirus salmonis* (Krøyer, 1838), in naturally inefcted salmonids and immunized rainbow trout, Oncorhynchus mykiss (Walbaum), and rabbits. Fish Shellfish Immunol..

[10] Hordvik I. (2015). Immunoglobulin isotypes in Atlantic salmon, Salmo Salar. Biomolecules.

[11] Xu Z., Parra D., Gómez D., Salinas I., Zhang Y.A., von Gersdorff Jørgensen L., Heinecke R.D., Buchmann K., LaPatra S., Sunyer J.O. (2013). Teleost skin, an ancient mucosal surface that elicits gut-like immune responses. Proc. Natl. Acad. Sci. U. S. A..

[12] Chen K., Xu W., Wilson M., He B., Miller N., Bengten E., Edholm E.-S., Santini P., Rath P., Chiu A., Cattalini M., Litzman J., Bussel J., Huang B., Meini A., Riesbeck K., Cunningham-Rundles C., Plebani A., Cerutti A. (2009). Immunoglobulin D enhances immune surveillance by activating antimicrobial, proinflammatory and B cell-stimulating programs in basophils. Nat. Immunol..

[13] Edholm E.-S., Bengten E., Stafford J., Sahoo M., Taylor E., Miller N., Wilson M. (2010). Identification of two IgD+ B cell populations in channel catfish, Ictalurus punctatus. J. Immunol..

[14] Danilova N., Bussmann J., Jekosch K., Steiner L.A. (2005). The immunoglobulin heavy-chain locus in zebrafish: identification and expression of a previously unknown isotype, immunoglobulin. Z. Nat Immunol.

[15] Hansen J.D., Landis E.D., Phillips R.B. (2005). Discovery of a unique Ig heavy-chain isotype (IgT) in rainbow trout: implications for a distinctive B cell developmental pathway in teleost fish. Proc. Natl. Acad. Sci. U. S. A..

[16] Zhang Y.A., Salinas I., Li J., Parra D., Bjork S., Xu Z., LaPatra S.E., Bartholomew J., Sunyer J.O. (2010). IgT, a primitive immunoglobulin class specialized in mucosal immunity. Nat. Immunol..

[17] Xu Z., Takizawa F., Casadei E., Shibasaki Y., Ding Y., Sauters T.J.C., Yu Y., Salinas I., Sunyer J.O. (2020). Specialization of mucosal immunoglobulins in pathogen control and microbiota homeostasis occurred early in vertebrate evolution. Sci. Immunol..

[18] Dong F., Yin G.-m., Meng K.-f., Xu H.-y., Liu X., Wang Q.-c., Xu Z. (2020). IgT plays a predominant role in the antibacterial immunity of Rainbow trout olfactory organs. Front. Immunol..

[19] Xu Z., Takizawa F., Parra D., Gómez D., von Gersdorff Jørgensen L., LaPatra S.E., Sunyer J.O. (2016). Mucosal immunoglobulins at respiratory surfaces mark an ancient association that predates the emergence of tetrapods. Nat. Commun..

[20] Van Muiswinkel W.B., Wiegertjes G. (1997). Immune responses after injection-vaccination of fish. Dev. Biol. Stand..

[21] Evensen Ø. (2016). Fish Vaccines.

[22] Mutoloki S., Munang’andu H.M., Evensen Ø. (2015). Oral vaccination of fish – antigen preparations, uptake, and immune induction. Front. Immunol..

[23] Matsuura Y., Takaoka N., Miyazawa R., Nakanishi T. (2017). A simple and non-invasive method for analyzing local immune responses in vivo using fish fin. Dev. Comp. Immunol..

[24] Shiota K., Sukeda M., Prakash H., Kondo M., Nakanishi T., Nagasawa T., Nakao M., Somamoto T. (2021). Local immune responses to two stages of Ichthyophthirius multifiliis in ginbuna crucian carp. Fish Shellfish Immunol..

[25] Salinas I. (2015). The mucosal immune system of teleost fish. Biology (Basel).

[26] Bui S., Oppedal F., Nola V., Barrett L.T. (2020). Where art thou louse? A snapshot of attachment location preferences in salmon lice on Atlantic salmon hosts in sea cages. J. Fish. Dis..

[27] Nortvedt R., Dahl-Paulsen E., Bizama L.P.A., Johny A., Slinde E. (2025). The effect of a polypeptide based vaccine on fish welfare and infestation of salmon lice, Lepeophtheirus salmonis, in sea cages with Atlantic salmon (Salmo Salar L.). Fishes.

[28] Tartor H., Karlsen M., Skern-Mauritzen R., Monjane A.L., Press C.M., Wiik-Nielsen C., Olsen R.H., Leknes L.M., Yttredal K., Brudeseth B.E., Grove S. (2021). Protective immunization of Atlantic salmon (Salmo Salar L.) against salmon lice (Lepeophtheirus salmonis) infestation. Vaccines.

[29] Rodríguez A., Gadan K., Pérez L., Evensen Ø., Estrada M.P., Carpio Y. (2025). Prime-boost vaccination with chimeric antigens adjuvanted in montanide™ ISA50 V2 confers protection against experimental Lepeophtheirus salmonis infestation in Atlantic salmon (Salmo Salar L.). Front. Immunol..

[30] Gislefoss E., Abdelrahim Gamil A.A., Øvergård A.-C., Evensen Ø. (2023). Identification and characterization of two salmon louse heme peroxidases and their potential as vaccine antigens. iScience.

[31] Hamre L.A., Glover K.A., Nilsen F. (2009). Establishment and characterisation of salmon louse (Lepeophtheirus salmonis (krøyer 1837)) laboratory strains. Parasitol. Int..

[32] Buchmann K. (2020). Immune response to Ichthyophthirius multifiliis and role of IgT. Parasite Immunol..

[33] Piazzon M.C., Galindo-Villegas J., Pereiro P., Estensoro I., Calduch-Giner J.A., Gómez-Casado E., Novoa B., Mulero V., Sitjà-Bobadilla A., Pérez-Sánchez J. (2016). Differential modulation of IgT and IgM upon parasitic, bacterial, viral, and dietary challenges in a perciform fish. Front. Immunol..

[34] Swain J.K., Carpio Y., Johansen L.-H., Velazquez J., Hernandez L., Leal Y., Kumar A., Estrada M.P. (2020). Impact of a candidate vaccine on the dynamics of salmon lice (Lepeophtheirus salmonis) infestation and immune response in Atlantic salmon (Salmo salar L.). PLoS One.

[35] Braden L.M., Monaghan S.J., Fast M.D. (2020). Salmon immunological defence and interplay with the modulatory capabilities of its ectoparasite Lepeophtheirus salmonis. Parasite Immunol..

[36] Fast M.D. (2014). Fish immune responses to parasitic copepod (namely sea lice) infection. Dev. Comp. Immunol..

