# Supplementary material for: Vaccination modality and injection site influence the immune response and protection against sea lice Lepeophtheirus salmonis infestation in Atlantic salmon (Salmo salar)
Source: Biochem Biophys Rep. 2026 Apr 8;46:102573. doi: 10.1016/j.bbrep.2026.102573 (PMC13091216; doi:10.1016/j.bbrep.2026.102573)
Supplement: Multimedia component 1 [file mmc1.docx]

Supplementary Table 1.

IgM/IgT combined in plasma and mucus by vaccine group (PBS reference). The differences between the recombinant and WSL vaccine groups versus PBS are shown.

|  | Plasma | | | | Mucus | | | |
| --- | --- | --- | --- | --- | --- | --- | --- | --- |
| Vaccine | **N** | **Mean (95% CI)** | **Δ vs PBS (95% CI)** | **P-values** | **N** | **Mean (95% CI)** | **Δ vs PBS mucus (95% CI)** | **P-values** |
| PBS | 15 | 0.051 (0.036 – 0.066) | 0 (ref) |  | 15 | -0.004 (-0.007 – -0.001) | 0 (ref) |  |
| IP | 15 | 1.427 (1.374 – 1.480) | 1.376 (1.322 – 1.430) | <0.001 | 15 | 0.143 (0.047 – 0.239) | 0.147 (0.052 – 0.241) | 0.003 |
| IP-F | 15 | 1.269 (1.151 – 1.386) | 1.218 (1.101 – 1.334) | <0.001 | 15 | 0.078 (-0.006 – 0.161) | 0.081 (-0.001 – 0.163) | 0.056 |
| LsPxtl-1 | 15 | 0.796 (0.588 – 1.004) | 0.745 (0.539 – 0.950) | <0.001 | 15 | 0.007 (0.000 – 0.015) | 0.011 (0.003 – 0.019) | 0.007 |
| LsPxtl-1+2 | 15 | 0.347 (0.238 – 0.457) | 0.296 (0.188 – 0.405) | <0.001 | 15 | 0.009 (-0.003 – 0.021) | 0.012 (0.000 – 0.025) | 0.053 |
| LsPxtl-2 | 15 | 0.621 (0.491 – 0.750) | 0.569 (0.441 – 0.698) | <0.001 | 15 | 0.004 (-0.004 – 0.011) | 0.007 (-0.000 – 0.015) | 0.061 |

IgT in plasma and mucus by vaccine group (PBS reference). The differences between the recombinant and WSL vaccine groups versus PBS are shown.

|  | Plasma | | | | Mucus | | | |
| --- | --- | --- | --- | --- | --- | --- | --- | --- |
| Vaccine | **N** | **Mean (95% CI)** | **Δ vs PBS (95% CI)** | **P-values** | **N** | **Mean (95% CI)** | **Δ vs PBS mucus (95% CI)** | **P-values** |
| PBS | 15 | 0.261 (0.221 – 0.302) | - |  | 9 | 0.003 (-0.001 – 0.008) | - |  |
| IP | 15 | 0.956 (0.909 – 1.002) | 0.695 (0.634 – 0.755) | <0.001 | 9 | -0.002 (-0.010 – 0.007) | -0.005 (-0.014 – 0.004) | 0.305 |
| IP-F | 15 | 0.888 (0.808 – 0.968) | 0.627 (0.538 – 0.716) | <0.001 | 10 | 0.009 (-0.002 – 0.020) | 0.006 (-0.006 – 0.018) | 0.337 |
| LsPxtl-1 | 15 | 0.349 (0.273 – 0.424) | 0.088 (0.003 – 0.172) | 0.046 | 15 | -0.005 (-0.011– -0.000) | -0.009 (-0.015 – -0.002) | 0.015 |
| LsPxtl-1+2 | 15 | 0.310 (0.263 – 0.358) | 0.049 (-0.013 – 0.111) | 0.123 | 15 | -0.011 (-0.019 – -0.003) | -0.015 (-0.023 – -0.006) | 0.002 |
| LsPxtl-2 | 15 | 0.292 (0.264 – 0.319) | 0.030 (-0.018 – 0.079) | 0.222 | 15 | -0.013 (-0.019 – -0.007) | -0.016 (-0.024 – -0.009) | <0.001 |
